# Efficient amplification of self-gelling polypod-like structured DNA by rolling circle amplification and enzymatic digestion

**DOI:** 10.1038/srep14979

**Published:** 2015-10-14

**Authors:** Tomoya Yata, Yuki Takahashi, Mengmeng Tan, Kumi Hidaka, Hiroshi Sugiyama, Masayuki Endo, Yoshinobu Takakura, Makiya Nishikawa

**Affiliations:** 1 Department of Biopharmaceutics and Drug Metabolism, Graduate School of Pharmaceutical Sciences, Kyoto University, Sakyo-ku, Kyoto, 606-8501, Japan; 2 Institute for Integrated Cell-Material Sciences (WPI-iCeMS), Kyoto University, Sakyo-ku, Kyoto 606-8501, Japan; 3 Department of Chemistry, Graduate School of Science, Kyoto University, Sakyo-ku, Kyoto 606-8502, Japan

## Abstract

The application of DNA as a functional material such as DNA hydrogel has attracted much attention. Despite an increasing interest, the high cost of DNA synthesis is a limiting factor for its utilization. To reduce the cost, we report here a highly efficient amplification technique for polypod-like structured DNA (polypodna) with adhesive ends that spontaneously forms DNA hydrogel. Two types of polypodna with three (tripodna) and four (tetrapodna) pods were selected, and a template oligodeoxynucleotide, containing a tandem sequence of a looped tripodna or tetrapodna, respectively, along with restriction enzyme (TspRI) sites, was designed. The template was circularized using T4 DNA ligase, and amplified by rolling circle amplification (RCA). The RCA product was highly viscous and resistant to restriction digestion. Observation under an electron microscope revealed microflower-like structures. These structures were composed of long DNA and magnesium pyrophosphate, and their treatment with EDTA followed by restriction digestion with TspRI resulted in numerous copies of polypodna with adhesive ends, which formed a DNA hydrogel. Thus, we believe this technique provides a new approach to produce DNA nanostructures, and helps in expanding their practical applications.

DNA has attracted much attention as a promising biomaterial because the polymer is natural, biocompatible, and biodegradable. It also has the ability to form designable nanostructures via its sequence-directed hybridization^1^,^2^. The progress in the field of DNA nanotechnology has demonstrated that a polypod-like branched DNA structure is one of the key building blocks for DNA nanoconstructions^3^,^4^,^5^. Recently, we reported that a polypod-like structured DNA, or polypodna, can be a potent immunomodulatory agent depending on the base sequence, as well as a component of an injectable self-gelling material for drug delivery^6^,^7^,^8^,^9^,^10^.

Synthesis cost is recognized as a major limiting factor for the application of DNA as a biomaterial^11^. It is because DNA nanostructures are typically made of chemically synthesized long single-stranded oligodeoxynucleotides (30–100 nt). Enzymatic synthesis is an attractive alternative, because these molecules quickly copy and amplify any template sequence with low error rates. Rolling circle amplification (RCA) is an enzymatic DNA amplification process that produces long single strands of the tandem-repeating sequence, which is complementary to the circularized single-stranded DNA template. Phi29 DNA polymerase, obtained from *Bacillus subtilis* phage phi29, possesses the functions not only for quick generation of polynucleotides, but also for strand displacement and proofreading under isothermal conditions. There have been some successful reports of Phi29 DNA polymerase-based amplification of simple DNA, such as linear oligodeoxynucleotide and DNA aptamer^12^,^13^,^14^,^15^, or semi-large scale amplification of complicated DNA structures^16^. However, no attempts have been made on mass production of more complex DNA structures such as self-gelling polypodna that spontaneously forms DNA hydrogel under proper conditions.

Here, we propose a highly efficient production technique for self-gelling polypodna by using RCA-based amplification. First, tripod-like structured DNA, or tripodna, was selected as a DNA nanostructure model and amplified. We designed a single-stranded template oligodeoxynucleotide consisting of the tandem sequence of a looped tripodna. This template also contained restriction enzyme sites so that tripodna units could be obtained by restriction digestion. Tetrapod-like structured DNA, or tetrapodna, was also prepared using two templates, each of which contained a half sequence of the tetrapodna. We found that this RCA-based technique can greatly increase the production efficiency of polypodna and reduce the synthesis cost, which will greatly increase the possibility of future application of such branched DNA nanostructures.

## Results

### RCA-based amplification of tripodna

**Figure 1:**
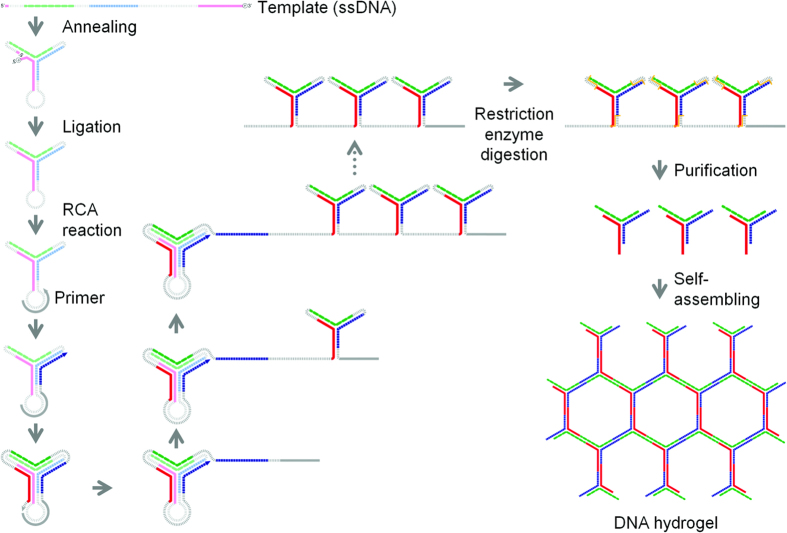
Schematic diagram of the mass amplification of tripodna with adhesive 5′-ends. The template oligodeoxynucleotides (template 1) were designed to satisfy the following requirements: (**a**) the polypodna automatically forms by self-assembly; (**b**) each pod of the polypodna contains a 9 base long TspRI restriction digest site; (**c**) Each 5′-terminal end is phosphorylated in order to ligate with 3′-terminal within the polypodna body, and (**d**) connecting chain is added to the 3′-terminal of the polypodna to allow polypodna to be connected to one another. The designed templates were amplified via the following steps. (**1**) The template ssODNs were circularized using T4 DNA ligase. (**2**) After annealing the primer (primer 1), the DNA template was amplified through rolling circle amplification technique using Phi29 polymerase. (**3**) Before enzyme digestion, the RCA product was treated with EDTA and folded. (**4**) Long single-stranded DNAs were digested using restriction enzyme. (**5**) The target sequences were purified by size chromatography. (**6**) The resultant DNAs self-assembled after annealing, and they formed a hydrogel.

**Figure 2:**
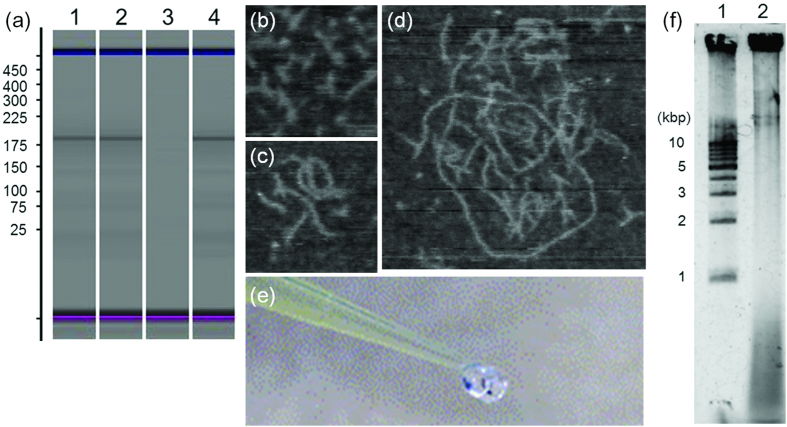
(**a**) Chip analysis of tripodna amplification. Lane 1, non-ligated template; lane 2, ligated template; lane 3, non-ligated template digested by exonuclease I/III; lane 4, ligated template digested by exonuclease I/III. (**B–D**) AFM imaging of the RCA products. (**b**) 0 h (before initiation of the RCA reaction), (**c**) 1 h, (**d**) 4 h. (**e**) RCA product after 16-h reaction. (**f**) Agarose gel analysis of RCA product. Lane 1, 1-kbp ladder; lane 2, RCA product.

Figure 1 illustrates the scheme of the RCA-based amplification. Figure 2(a) shows the microchip electrophoresis of the template before and after ligation using T4-ligase. There was no significant difference in the electrophoretic mobility of the template before and after ligation (Fig. 2(a), lanes 1, 2). This could be due to that the template would be folded in a similar structure to the ligated, circularized one. To confirm the ligation, the non-ligated and ligated templates were digested by exonuclease I/III (Fig. 2(a), lanes 3, 4). Only the ligated template was resistant to the digestion (Fig. 2(a), lane 4), indicating that the template was circularized by T4-ligase. After exonuclease digestion of the non-circularized template oligonucleotides, the circularized template was replicated by RCA. The AFM images of the template and products of the RCA reaction showed that the RCA products were elongated with time (Fig. 2(b–d)). A long reaction time resulted in highly viscous products as shown in Fig. 2(e). Agarose gel electrophoresis showed that the template was largely amplified (Fig. 2(f), lane 2).

**Figure 3:**
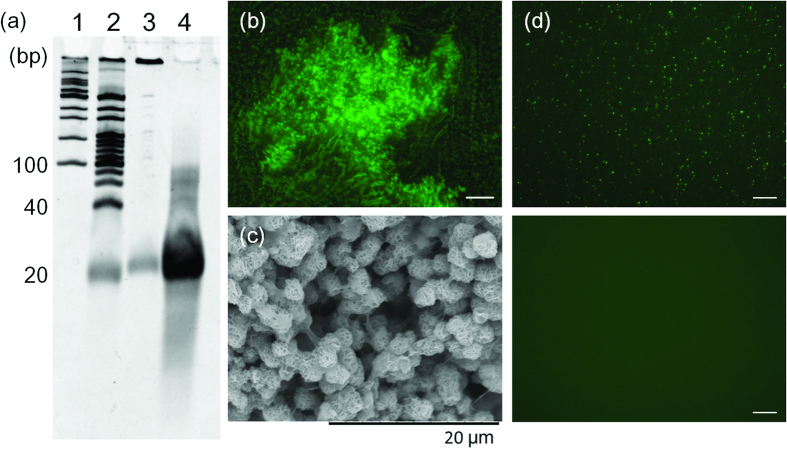
(**a**) Denaturing PAGE analysis of RCA products before and after digestion by restriction enzyme. Lane 1, 100 p marker; lane 2, 20 bp marker; lane 3, digested DNA fragments without EDTA treatment; lane 4, digested DNA fragments after EDTA treatment. (**b**) Fluorescent microscope image of the RCA product. The highly viscous RCA product was stained using SYBR Gold. Scale bar = 100 μm. (**c**) SEM image of the RCA product. RCA product was dehydrated using ethanol, and freeze-dried. Particle size of microflower like structure was estimated around 2–3 μm, and similar to the structure of RNAi-microsponges previously reported^19^. (**d**) Fluorescent microscope image of RCA product before/after treatment of EDTA. (upper) Before EDTA treatment, microparticles were observed. (lower) After EDTA treatment, microparticles disappeared. Scale bar = 50 μm.

Then, the RCA product was purified and digested with TspRI, and the digested sample was analyzed by denaturing PAGE (Fig. 3(a)). The digestion generated only a small amount of short ODN products (Fig. 3(a), lane 3). To understand the cause of this unsuccessful digestion, the structure of the RCA product was visualized under a fluorescent microscope. Staining the RCA product with SYBR Gold revealed that the RCA product contained many microparticles (Fig. 3(b)). Observation under a SEM showed that the microparticles were in microflower-like structures (Fig. 3(c)). It has been reported that RCA products are densely packed and resistant to enzyme digestion^17^,^18^. Breaking down the microflower-like structure of the RCA product would be useful for the efficiency of its restriction digestion. Extensive studies on the microstructure of interfering RNAs, (RNAi)-microsponges produced by T7-RNA polymerase, have shown that the RNA microstructure was composed of magnesium pyrophosphate, and it was capable of being denatured by EDTA^19^. Pyrophosphate is a side product of the nucleotide coupling reaction, and it is produced in RCA. Therefore, it was assumed that the DNA microflower-like structure generated by the RCA reaction is also composed of magnesium pyrophosphate, and is able to be denatured by EDTA. As expected, the addition of EDTA broke up the microparticles in the RCA reaction solution (Fig. 3(d)). After purification of polynucleotides by size-exclusion chromatography, the solution was heated to 95 °C and cooled gradually down to 4 °C to form tripodna into the long chain polynucleotides for restriction enzyme digestion. In this case, the polynucleotides were efficiently digested into short fragments. Denaturing PAGE analysis clearly showed that oligonucleotides were efficiently produced by restriction digestion of the RCA products after EDTA treatment (Fig. 3(a), lane 4).

**Figure 4:**
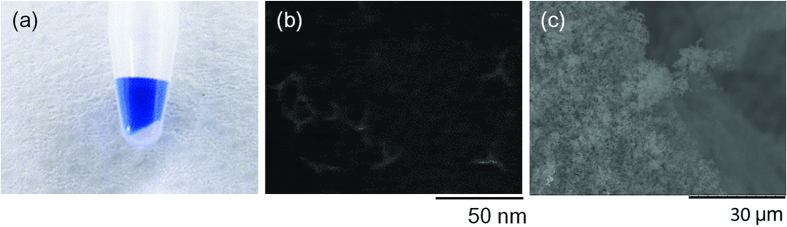
(**a**) Optical image of resulting DNA hydrogel. The solution containing blue dextran (Sigma Aldrich, St. Louis, MO, USA) was added to check the hydrogel formation. Blue dextran did not instantly diffuse into hydrogel. (**b**) AFM image of the RCA product obtained using the tripodna template. (**c**) SEM image of resulting DNA hydrogel.

The oligonucleotides were purified by size-exclusion chromatography, and annealed at 95 °C. The products were not capable of being mixed with a solution containing blue dextran (Sigma-Aldrich, St. Louis, MO, USA) (Fig. 4(a)), which confirmed the formation of a hydrogel. AFM imaging showed that the products self-assembled into oligomers or multimers under the diluted conditions (Fig. 4(b)). Observation under SEM (Fig. 4(c)) showed that the inner structure of the hydrogel obtained by this technique was comparable to that of the DNA hydrogel made up of tripodna with chemically synthesized oligodeoxynucleotides reported previously^20^. These results suggest that the DNA hydrogel is formed by self-assembly of the tripodnas through the sticky ends. In this synthesis, 0.5 nmol of template was amplified successfully by approximately 300 fold to produce 150 nmol of tripodna.

### RCA-based amplification of tetrapodna

**Figure 5:**
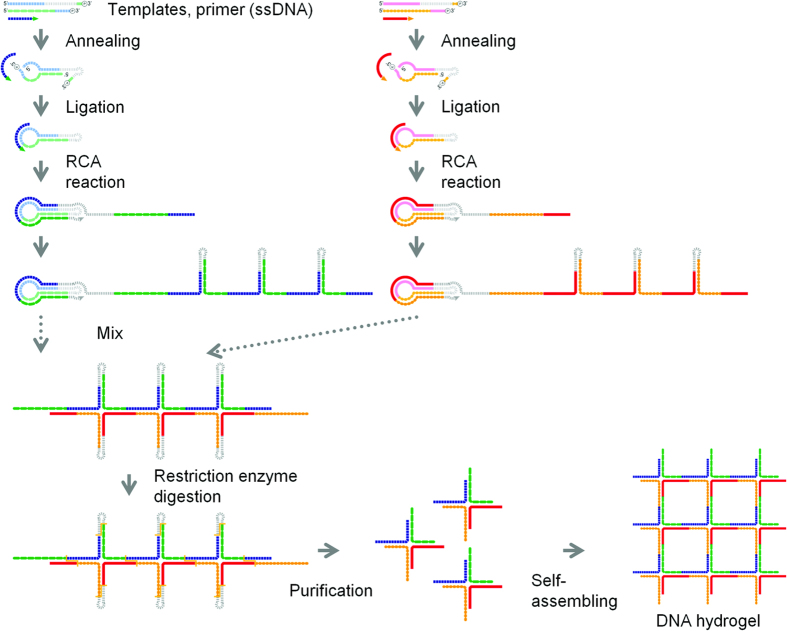
Schematic diagram of the mass amplification of tetrapodna with adhesive 5′-ends. Short fragment templates (template 2–1, template 2–2, template 3–1, and template 3–2) were used to reduce cost, and two different circular templates were constructed using these fragment templates and primers (primer 2 and primer 3). Except for these, the same protocol as described in the legend of Fig. 1 was used to amplify the templates.

**Figure 6:**
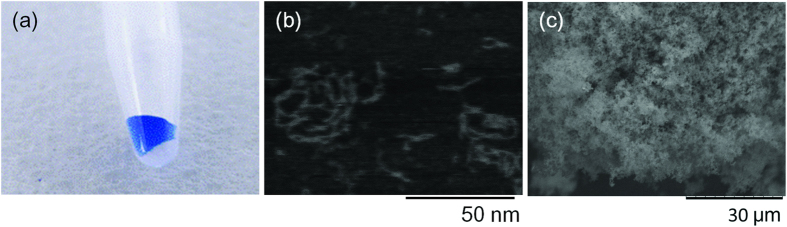
(**a**) Optical image of the resulting DNA hydrogel. The solution containing blue dextran (Sigma Aldrich, St. Louis, MO, USA) was added to check the hydrogel formation. (**b**) AFM image of the RCA product obtained using the tetrapodna templates. (**c**) SEM image of resulting DNA hydrogel.

To expand this RCA-based amplification to more complicated DNA nanostructures, tetrapodna was selected and amplified by the same procedure, as schematically described in Fig. 5. The template of a tetrapodna was longer than that of a tripodna, because a tetrapodna unit contains more nucleotides than a tripodna unit. To avoid the costliness and technical difficulty associated with the preparation of a long template, we separated a long template sequence for a tetrapodna into two short sequences. The two templates for a tetrapodna were used for the RCA-based amplification. The two templates were separately amplified through rolling circle amplification. After treatment with EDTA, the two RCA products were digested with TspRI, which produced 60 base oligodeoxynucleotides. The mixture resulted in the formation of a DNA hydrogel (Fig. 6(a)) in the same manner as tripodna. Oligomers and multimers of tetrapodna units were observed under AFM imaging in dilute solutions (Fig. 6(b)). The inner structure of the hydrogel was similar to that of a tetrapodna-based hydrogel consisting of chemically synthesized ODNs (Fig. 6(c)). Again, approximately a 300-fold amplification was achieved to obtain 150 nmol tetrapodna from 0.5 nmol of template.

## Discussion

We demonstrated in the present study that tripodna and tetrapodna, two DNA nanostructures that are effective for the delivery of immunostimulatory DNA to immune cells, can be efficiently replicated in large quantities (approximately 300-fold) by RCA. This technique requires basic biochemical laboratory equipment and basic reagents, and can be performed with little technical difficulty. Although TspRI was used to create a nine nucleotide sticky-ends to produce self-gelling polypodna, other restriction enzymes can also be used. The principle underlying this approach can be easily applied to replicate other designs of polypodna and many other complex DNA nanostructures. There have been some reports that apply the RCA technique to the amplification of DNA nanostructures^12^,^13^,^14^,^15^,^16^, but none of them mentioned unsuccessful enzyme digestion of the RCA products or the high viscosity of the RCA reaction solution. It might be because previous studies were conducted with low concentrations of DNA. In our study, rolling circle amplification was performed with a high DNA concentration to produce short ODN products for DNA nanostructures efficiently. The present study indicates for the first time that the degradation of the byproduct in the RCA reaction using EDTA enables us to increase the DNA concentration for RCA reaction. Therefore, the scheme that we describe here can provide a solution to overcome the major obstacle for large-scale production of DNA nanostructures.

The DNA nanostructures covered in this study were relatively simple ones, i.e., tripodna and tetrapodna. However, as we proposed and demonstrated here, our technique can be expanded, and applied to complex DNA nanostructures. These include hexapodna, truncated octahedrons^21^, octahedrons^22^, tetrahedrons^23^, and DNA buckyballs^24^. The results of the tetrapodna directly showed that the use of two or more templates can increase the complexity of DNA nanostructures without additional difficulties in both design and production.

In our proof-of-concept study, the scale of the final products was in microgram scale, and there are still challenges to scale up the reaction volume for practical application. However, we consider it useful to provide an alternative method to chemical synthesis for efficient amplification of such complex structured DNA building blocks as polypodna for expanding the application of DNA hydrogel. The overall costs for the preparation of tripodna and tetrapodna were less than those required for the ODNs for tripodna and tetrapodna, even under the conditions used for the small-scale study, although a strict comparison between them is difficult due to the large difference in the price for ODNs. Because the cost for phi29 DNA polymerase accounted for a significant proportion (more than 70%) of the total cost in the present study, optimization of the RCA reaction would greatly reduce the total cost of our method.

In conclusion, we developed for the first time an efficient synthesis method for self-gelling polypod-like structured DNA that spontaneously forms DNA hydrogel under proper conditions. We believe that this technique provides a new approach to amplify DNA nanostructures, and helps in expanding their practical applications.

## Methods

### Preparation of templates

All oligodeoxynucleotides were purchased from Integrated DNA Technologies, Inc (Coralville, IA, USA). The sequences of the oligodeoxynucleotides used are summarized in Table 1. A linear 5′-phosphorylated template oligodeoxynucleotide, template ODN (50 μM), was self-annealed by heating at 95 °C for 2 min, 75 °C for 3 min, then gradually cooled down to 4 °C. The annealed oligodeoxynucleotide was ligated at 16 °C for 16 h in solution containing 10 U/μL T4 DNA ligase (Takara Bio, Otsu, Japan), 66 mM Tris-HCl (pH 7.6), 6.6 mM MgCl_2_, 10 mM dithiothreitol (DTT), and 0.1 mM ATP. Non-circularized linear oligonucleotides were removed by reaction with 25 U/mL exonuclease I (Takara Bio) and 1000 U/mL exonuclease III (Takara Bio) at 37 °C for 30 min.

### RCA-based amplification of polypodna precursors

An RCA primer was designed to hybridize to the single stranded sequence of the circularized template. Equivalent amounts of the circularized template and the primer were mixed together, and these were annealed under the above conditions. The resultant mixture (10 μM) was amplified by incubating at 30 °C for 16 h in a solution containing 2.5 U/μL phi29 DNA polymerase (New England Biolabs, Ipswich, MA, USA), 50 mM Tris-HCl (pH 7.5), 10 mM MgCl_2_, 10 mM (NH_4_)_2_SO_4_, 4 mM DTT, 200 μg/ml BSA, and 2.5 mM dNTP (Invitrogen, Carlsbad, CA, USA).

### Polypodna production by restriction digestion

The highly viscous RCA product was incubated in 2 mM EDTA (Sigma-Aldrich, St. Louis, MO, USA) at 80 °C for 15 min to solubilize the product. After purification by size-exclusion chromatography, the resultant large molecular weight DNA was digested with 0.1 U/μL TspRI (New England Biolabs) in solution containing 50 mM potassium acetate, 20 mM Tris-acetate, 10 mM magnesium acetate, and 100 μg/ml BSA. The product was purified by size-exclusion chromatography to remove low molecular weight DNA waste. Restriction digestion with TspRI was performed at 50 °C, which was determined to be the optimal temperature for digestion with TspRI, based on preliminary experiments.

### Polypodna formation

The RCA product obtained after restriction digestion was annealed as reported previously^8^. Briefly, the 1.5- or 2 mM-DNA products for tripodna or tetrapodna, respectively, were heated to 95 °C and cooled gradually to 4 °C. The formation of hydrogels were observed optically using blue dextran solution as previously reported^9^.

### Oligonucleotide analysis

DNA products in each step were analyzed using a MCE-202 MultiNA microchip electrophoresis system (Shimadzu Corporation, Kyoto, Japan).

### Observation of RCA product under fluorescent microscope

The appearance of the RCA product before and after EDTA treatment was observed under a fluorescent microscope (Biozero BZ-8000, KEYENCE, Osaka, Japan) after staining with SYBR-Gold (Molecular Probes, Eugene, OR, USA).

### Scanning electron microscope imaging

The RCA product obtained after restriction digest was annealed under the above conditions. The structure of the annealed RCA products was then observed using a scanning electron microscope (TM3000, Hitachi, Tokyo, Japan) as reported previously^8^.

### Atomic force microscope imaging

Atomic force microscope images were obtained with a high-speed AFM system (Nano Live Vision, RIBM, Tsukuba, Japan) using a silicon nitride cantilever (BL-AC10EGS; Olympus, Tokyo, Japan)^25^. Briefly, the sample was adsorbed on a freshly cleaved mica plate pretreated with 0.1% aqueous 3-aminopropyltriethoxysilane for 5 min at room temperature and then washed three times with a buffer solution containing 20 mM Tris and 10 mM MgCl_2_. To observe the elongation of the RCA products, aliquots were sampled at 0 (before initiation of the reaction), 1, and 4 h after the onset of the RCA reaction. Then, the collected samples were heated to 95 °C to halt the reaction, annealed, and diluted to a DNA concentration of 30 nM to avoid hydrogel formation. Then, the samples were observed by AFM as described above.

## Additional Information

**How to cite this article**: Yata, T. *et al*. Efficient amplification of self-gelling polypod-like structured DNA by rolling circle amplification and enzymatic digestion. *Sci. Rep*. **5**, 14979; doi: 10.1038/srep14979 (2015).

## Figures and Tables

**Table 1:** Sequences of the oligonucleotides used in this study.

Role	Sequences
Tripodna	
Template 1	tgc gcc aat ggc aaa agc caa tgg cgc acg tcg tag tgc att gac agc gtc tag cta gcc aat ggc aaa agc caa tgg cta gct aga cgc tgt caa gca gac gtc gat caa gcc aat ggc aaa aaa aaa aaa aaa aaa aaa aaa aaa aaa aaa aaa aaa aaa gcc aat ggc ttg atc gac gtc tgc tat gca cta cga cg
Primer 1	ttt ttt ttt ttt ttt ttt ttt ttt t
Tetrapodna	
Template 2–1	cta gac cgt gtc atg acg ctc agc tgc aag cca ctg gct tcg aaa aaa aac gaa gcc ag
Template 2–2	tgg ctt gca gct gag cgt caa gca gac gtc gat caa gcc agt ggc ttg
Primer 2	cgt cta gca agc cac tgg ct
Template 3–1	atc gac gtc tgc tgc acg tcg tag tgc aag cca gtg gct tcg aaa aaa aac gaa gcc ac
Template 3–2	tgg ctt gca cta cga cgt gct gac agc gtc tag caa gcc act ggc ttg
Primer 3	agc aga cgt cga tca agc cag tgg ct
