# Corrigendum: Efficient amplification of self-gelling polypod-like structured DNA by rolling circle amplification and enzymatic digestion

**DOI:** 10.1038/srep17249

**Published:** 2016-01-07

**Authors:** Tomoya Yata, Yuki Takahashi, Mengmeng Tan, Kumi Hidaka, Hiroshi Sugiyama, Masayuki Endo, Yoshinobu Takakura, Makiya Nishikawa

Scientific Reports 5: Article number: 1497910.1038/srep14979; published online: 10142015; updated: 01072016.

In this Article, Affiliations 2 and 3 are not listed in the correct order. The correct affiliations are listed below:

Affiliation 2

Department of Chemistry, Graduate School of Science, Kyoto University, Sakyo-ku, Kyoto 606-8502, Japan

Affiliation 3

Institute for Integrated Cell-Material Sciences (WPI-iCeMS), Kyoto University, Sakyo-ku, Kyoto 606-8501, Japan

